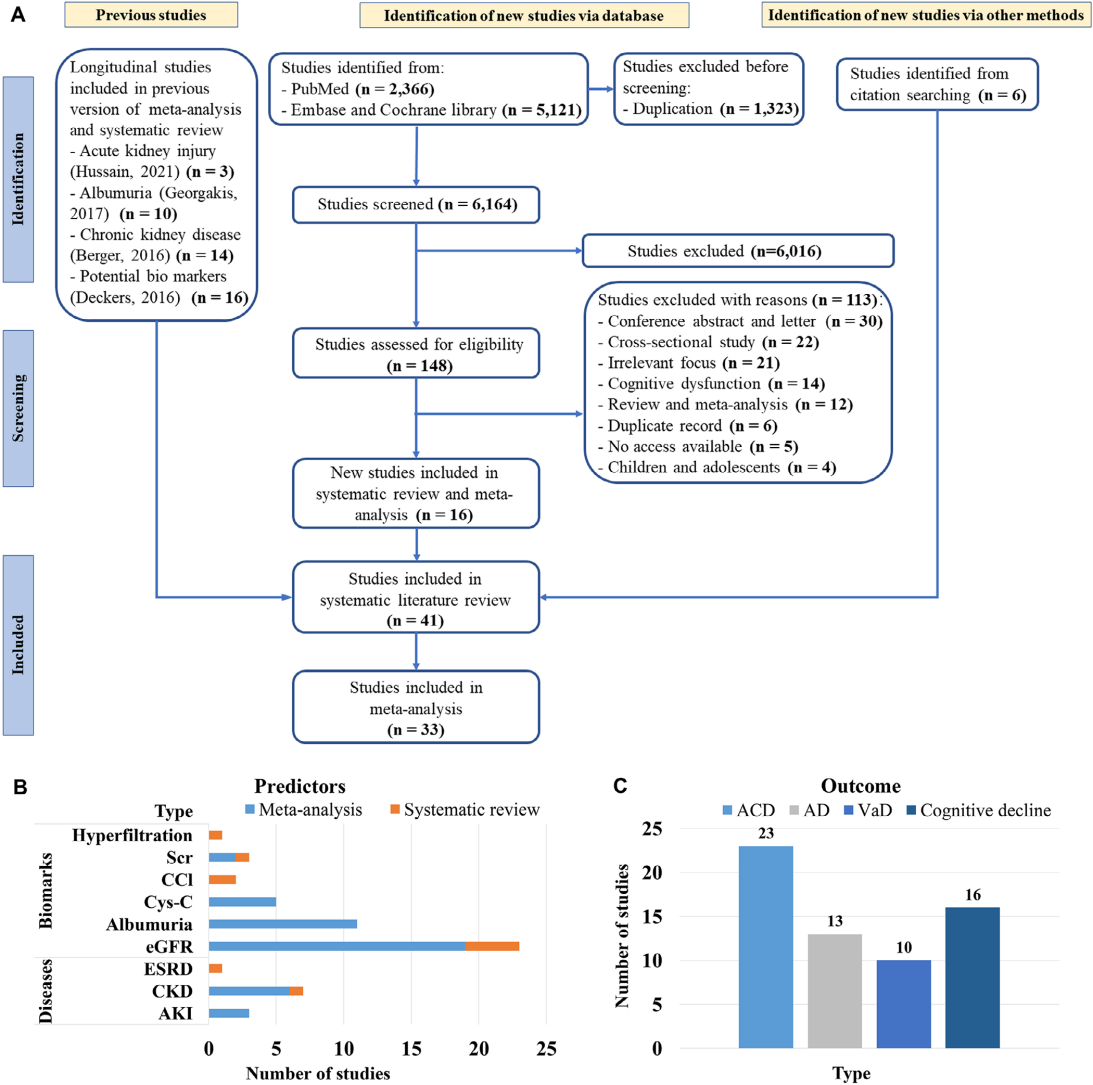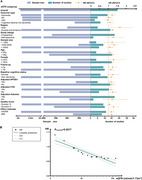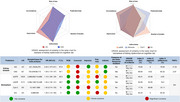# Adult Renal Dysfunction and Risk of Dementia or Cognitive Decline: Brain‐Kidney Axis Hypothesis Based on a Systematic Review and Meta‐Analysis

**DOI:** 10.1002/alz70860_101578

**Published:** 2025-12-23

**Authors:** Hao‐chen Chi, Lan Tan

**Affiliations:** ^1^ Qingdao Municipal Hospital, Qingdao, Shandong, China; ^2^ Qingdao Municipal hospital, Qingdao university, Qingdao, Shandong, China

## Abstract

**Background:**

The brain‐kidney axis was proposed to emphasize roles of kidney functioning in modulating neurodegeneration.

**Method:**

The PubMed, EMBASE, and Cochrane library were searched until February 1st, 2022, to include longitudinal studies. Multivariate adjusted effects were pooled by random‐effects models. The robust error meta‐regression models were used for dose–response analyses. The credibility of meta‐analyses was graded and an innovative index (S_difference_) was developed to evaluate the evidence tendency.

**Result:**

A total of 41 longitudinal studies (6,480,136 participants, mean age range: 58.5‐83.5 years) were included, of which 33 were for meta‐analyses. Though with low level of evidence, five indicators of kidney were associated with increased risk of dementia or cognitive decline, including acute kidney injury (hazard ratio [HR] = 2.24, *p* = 0.0001), chronic kidney disease (HR = 1.29, *p* = 0.0001), higher serum creatinine (HR = 1.35, *p* = 0.0001), higher urine albumin creatine ratio (UACR, HR = 1.23, *p* = 0.0001), and lower estimated glomerular filtration rate (eGFR, HR = 1.18, *p* = 0.0001). A linear relationship was revealed for eGFR (*p* = 0.0217) or UACR (*p* = 0.0006). Heterogeneity is a main concern to jeopardize the evidence robustness, especially for eGFR (S_difference_ = 0.05).

**Conclusion:**

Some renal indicators were associated with a higher risk of dementia, though the evidence base warrants further strengthening. Renal function management might serve as a promising target for dementia prediction and prevention.